# Supplementary material for: Patterns of Intron Gain and Loss in Fungi
Source: PLoS Biol. 2004 Nov 30;2(12):e422. doi: 10.1371/journal.pbio.0020422 (PMC532390; doi:10.1371/journal.pbio.0020422)
Supplement: Table S1 — Also available at http://genes.mit.edu/NielsenEtAl/. (4.3 MB ZIP). [file pbio.0020422.st001.zip › NielsenEtAl/html/1380.html]

AN0608.1.NCU06062.1.MG08289.1.FG04334.1


```
 CLUSTAL W (1.82) Multiple Sequence Alignments - Introns Inserted


Sequence 1: NCU06062.1	446 aa
Sequence 2: MG08289.1	448 aa
Sequence 3: FG04334.1	457 aa
Sequence 4: AN0608.1	443 aa
Alignment Length: 477 aa
Number Identitical Residues: 153 aa
Alignment Score (without introns) 8953


MG08289.1 	MPPP--ALSSLDS----STTVLRLPHPYHTPYAVTKSSRTHGSDELPLYQLGITSTAPAG
NCU06062.1	MASP--TTRRLAEYVANSEPILKLPHPYQTSYLVVKGEPGVANS----YQLKQQDSK-AA
FG04334.1 	MSTPSGTFSNLKA----GEIIVKLPHPYQTAYTIEAASTPLGVNTAVYKLVEKASVSAKA
AN0608.1  	MASAD------------KLPLIRLPEPYKTTYRLQVVSVQHGTR---KLQLRRSPRVDDG
          	*....               :::**.**:*.* :   .   .        :        .

MG08289.1 	LAADRASHAVLHSDGIFFTEPTDLKSSDRPHESNNTPWGRARRSPSVTALWDAKRSALPT
NCU06062.1	LPRP------LHNDSLFFTEPQDLKSSDKPHISINTPWGRARRSPSVAVSWKEP--SAPT
FG04334.1 	LPFN------PQHPSLVFSDPIDLKSSQLPSQSNNSPWARARRSPCVTVYWNEA--EAPT
AN0608.1  	LPPPEP----LHRDSFHFTDLREPDPYTTPPEGNNSAWARAQRSPVTYWNWEGQ--EPPT
          	*.   .     :  .: *::  : ..   *  . *:.*.**:*** .   *.      **

MG08289.1 	PAQLWLLAYLVFTVRPAEEAFRLTLNDGTSTAEGPRALGSRLKDLMLAVDHPVDSTDG--
NCU06062.1	VGQLWLIAYVIFTLRPSEEAFRLSTYG-----FGADKLARQLKAVGLAIEHPAPATSEQQ
FG04334.1 	LAQVWLLAYALFTVRTNVESYRLELRG-----VNASVLGQQLTAVLLAIDHPLKGREKRQ
AN0608.1  	VAQIWNVLYALLTLRTEFEIFRAVLSG-----EGRELLAQELQAVGLATSHPSPSAPPGQ
          	 .*:* : * ::*:*.  * :*    .      .   *. .*  : ** .**  .    .

MG08289.1 	---AAAPPSE--LLVLRSTFWQGAGSPFGPRPVWAPDEAGTSS--------YPLAPLDGT
NCU06062.1	KLHGSDAGKE--LLILRGAFWQGAGSPFGARPAWAPETLADSEDGS-LESVYPLQATEYV
FG04334.1 	---ASDKTDESLVLLLRSTFWQGAGSPFGPRPVWCPAESPASLPASNPLGSYPLTPLHNT
AN0608.1  	ILPQSAGTPD-QLVIYRSTFWQGAGSPFGSRPVWVCDPDIYTSLRR-PLTAFPVHPVQHT
          	    :    :  ::: *.:**********.**.*       :         :*: . . .

MG08289.1 	LTAIPG--------TALCWHPRRPAKPRPGSVVYSRYVPHLAETFSMVALDWTDDAHLGL
NCU06062.1	MTNEPN--------SALCWHPRRRAKPAPGSVIYSRYIPHLKENFSMVALDYTNPAHLEL
FG04334.1 	TTITVAGDPDDPERVQQSWHPVRPVKPAPGATVYSRWIPHLNETFSMVSLDYTDAEHVRL
AN0608.1  	LTTKTS------DVRVHALHPIRPRKPTPGSKMYSRYIPQLDEFFSLWALDYRDDEHLRL
          	 *          .    . ** *  ** **: :***::*:* * **: :**: :  *: *

MG08289.1 	FHAWQNDPRVSQGWNETGTLDQHREYLRRLHEDPHQFAVLAAFEGVFFAYFEIYWAK0ED
NCU06062.1	FHTWQNDPRVSQGWNEEGTLEQHREYLRKAHEDPHQITVLAAFDDVFFAYFEIYWAK0ED
FG04334.1 	FHEWQNDPRVSQGWNLTGTLKQHQEYLHKVHDDPHVIALLAKWNDTSFAYFEVYWAK0ED
AN0608.1  	FHMWQNDPRVARGWNETGTLEQHREYLRKLEEDPHQIAVLAKFNNTFFSYHQIYWAK0ED
          	** *******::***  ***.**:***:: .:*** :::** ::.. *:*.::**** **

MG08289.1 	RLGAYYQSGDFDRGRHSLVGDVRFRGPHRVSAWWSSLMHFLYLDEPRTTAVVGEPKSANS
NCU06062.1	RLGAYYTPGDYDRGRHSLVGDVRYRGPHRVSAWWSSLMHYLYLDDPRTMNVVGEPKYTNT
FG04334.1 	RLGGYFNAGDFDRGRHSLVGDVRFRGPHRVSAWWSSLMHYLYLDDPRTMFVVGETQETNT
AN0608.1  	SIGAHYDADDWDRGRHSLVGDVRFRGQHRVIVWWCSIMHYMFLDESRTRYIVGEPEFTNL
          	 :*.:: ..*:************:** *** .**.*:**:::**:.**  :***.: :* 

MG08289.1 	TVLSYDFIHGFGVEKFVDLPHKRSGLMHCSRERFFQLCPLAENDKVVGGTGVGLVPKL
NCU06062.1	SVLMYDMMHGFGLDKFVDLPHKRSAFVTCSRERFFQLCPLDENQKFLGGTHLGLVPKL
FG04334.1 	TIVMYDFVHGSGLDKLVDLPHQRSAFVRSSRERFFQLCPLADNEKVVAGTKLGLVPKL
AN0608.1  	TVLAYDHATGFGLEKLIDLPHKRSALVKCSREKFFQISPFRFDGSDHLERDPYRALKL
          	::: **   * *::*::****:**.:: .***:***:.*:  : .         . **
```
